# The effects of metabolic and functional traits on bud opening: Comparing warming and defoliation in conifers

**DOI:** 10.1093/plphys/kiaf435

**Published:** 2025-09-26

**Authors:** Annie Deslauriers, Pascale Benoit, Lorena Balducci, Valérie Néron, Rosario Guzmán-Marín, Sergio Rossi, Serge Lavoie, Nathalie Isabel

**Affiliations:** Centre de recherche sur la boréalie (CREB), Département des Sciences Fondamentales, Université du Québec à Chicoutimi, Chicoutimi, QC, Canada G7H 2B1; Centre de recherche sur la boréalie (CREB), Département des Sciences Fondamentales, Université du Québec à Chicoutimi, Chicoutimi, QC, Canada G7H 2B1; Centre de recherche sur la boréalie (CREB), Département des Sciences Fondamentales, Université du Québec à Chicoutimi, Chicoutimi, QC, Canada G7H 2B1; Bureau du Forestier en Chef, Ministère des Ressources naturelles et des Forêts, Roberval, QC, Canada G8H 2L6; Centre de recherche sur la boréalie (CREB), Département des Sciences Fondamentales, Université du Québec à Chicoutimi, Chicoutimi, QC, Canada G7H 2B1; Centre de recherche sur la boréalie (CREB), Département des Sciences Fondamentales, Université du Québec à Chicoutimi, Chicoutimi, QC, Canada G7H 2B1; Centre de recherche sur la boréalie (CREB), Département des Sciences Fondamentales, Université du Québec à Chicoutimi, Chicoutimi, QC, Canada G7H 2B1; Centre de recherche sur la boréalie (CREB), Département des Sciences Fondamentales, Université du Québec à Chicoutimi, Chicoutimi, QC, Canada G7H 2B1; Service canadien des forêts, Ressources naturelles Canada, Québec, QC, Canada G1V 4C7

## Abstract

Developing buds are crucial carbon sinks that require nonstructural carbohydrates (NSCs) for growth. However, the trade-off between carbon production in the older internodes and the demand in the growing internodes for bud opening remains unknown. Here, we determined how NSCs and functional traits influence bud phenology in the saplings of 2 conifer species. To manipulate both source and sink, saplings of balsam fir (*Abies balsamea*, L. Mill) and black spruce (*Picea mariana* B.S.P. (Mill.) were exposed to 2 simultaneous treatments: warming (+2 °C) and defoliation. Balsam fir, the species with earlier phenology, exhibited greater shoot volume and specific leaf area, promoting water and carbon acquisition for primary growth. Heating led to an earlier phenology but did not affect the leaf traits for both species. Defoliation also led to an earlier phenology, mostly because of the decreased growing sink, with fewer needles and smaller specific leaf area needed for growth. Starch and sucrose levels in older needles and growing buds decreased under defoliation, but the sugar alcohol D-pinitol remained unchanged. Heating increased the D-pinitol concentration in the growing buds (+17%) compared to ambient conditions. Under warming, a high D-pinitol concentration in buds can act as a carbon sink in the vacuole, maintaining or increasing water absorption, and thus, resulting in faster needle expansion and bud opening. These data demonstrate that different physiological mechanisms explain earlier bud opening under defoliation and warming. Additional studies are needed to disentangle the roles of leaf traits and carbon allocation in regulating phenology.

## Teaser

Warming and natural defoliation affect the development of balsam fir and black spruce buds differently in their sugar allocation as well as their functional traits and timing

## Introduction

The break of dormancy in the buds of perennial plants is characterized by morphological and physiological changes (Lipavská et al. 2000; Maurel et al. 2004; Xu et al. 2016). Bud meristems are the source of signal perception for dormancy release, responding not only to temperature and photoperiod but also to endogenous factors, such as phytohormones (Xu et al. 2016), sugars, and water (Pantin et al. 2012). The classical view holds that hormones influence bud and shoot development; however, an emerging perspective is that the interactions of source–sink relationships can trigger transition development in primary meristems. During bud development, variations in starch and soluble sugar concentrations and water content (Rinne et al. 1994) influence growth dynamics, i.e. phenology and the extension of shoots. The allocation and partitioning of sugars to the meristem are particularly important for bud outgrowth (Barbier et al. 2015), as the shoot apical meristem is a key part of the plant that uses carbon for primary growth (Bonhomme et al. 2010; Cartenì et al. 2023). Recently, Wingler and Henriques (2022) underlined that bud burst during the spring also depends on sucrose availability. However, there are very few studies on how resource availability, such as for sugar (i.e. metabolite pool), affects the process of bud opening and the timing of leafing and shoot growth (Lipavská et al. 2000; Deslauriers et al. 2019; Cartenì et al. 2023).

During spring, sugars influence new organ growth (e.g. buds) in plants, either by acting as substrates for newly synthesized components or by having a signaling role (Wingler 2018). Throughout bud swelling and leaf–shoot elongation, plants require an adequate carbon supply to sustain the metabolic demands until organ autotrophy is reached (Pantin et al. 2012). These nonstructural carbohydrates (NSCs) are composed of sugars and sugar alcohols providing both carbon for expansive and structural growth (Popp et al. 1997; Koch 2004; Pantin et al. 2012). Leaf expansion, more sensitive to water decrease, requires the formation of a carbon sink or sugar metabolites pool in the vacuole to attract water, along with other osmolytes, leading to osmotic adjustments (Turner 2018). Among the soluble sugars, sugar alcohols, such as D-pinitol in conifers, formed major constituents of plant-soluble components that can be transported into the phloem (Dumschott et al. 2017). Structural growth mainly requires sucrose that is break down by sucrose synthase to form cellulose (Koch 2004). How strong is the ability to import sugars reflects bud growth capacity, regulating at the same time stem/bud elongation (Salam et al. 2021). In conifers, sugars partitioned from nearby existing leaves are distributed to buds via the vascular system. Because of seasonal and environmental limitations at high latitudes, starch accumulation in boreal conifers begins at the end of winter. Starch concentrations increase until the beginning of bud opening, when this compound reaches its highest level at bud break in leaves and roots (Schadel et al. 2009; Deslauriers et al. 2019; Schoonmaker et al. 2021). At bud break, the starch reserves stored in the old leaves are transformed into sugars that are used for restarting growth and developing new shoots, adding to the new assimilates produced by both old and new foliage (Cartenì et al. 2023). These processes are mainly driven by abiotic conditions (Cartenì et al. 2023); however, the crucial role of sugar pathways in the sink–source effect for ensuring optimal stem development is not completely elucidated.

Warming is known to accelerate growth reactivation in spring, leading to an overall earlier bud break (Delpierre et al. 2016; Piao et al. 2019). Temperature is sensed locally within each bud (Vitasse et al. 2021), but how spring temperatures interact with the sugar supply to buds remains little studied (Blumstein et al. 2024). However, across different species, a greater sugar allocation to buds advances swelling, leading to an earlier bud opening and shoot growth (Savage and Chuine 2021). Indeed, adding sucrose promotes branching in potato (*Solanum tuberosum* L.) (Salam et al. 2021) and accelerates bud opening in *Rosa hybrida* (Barbier et al. 2015). However, biotic and abiotic factors can affect the available sugar concentration for growth. Among these, temperature warming is known to decrease the level of hexoses in leaves (Way and Sage 2008) and wood (Deslauriers et al. 2014) of conifers because of higher carbon loss through respiration at night (Dusenge et al. 2019), thus limiting sugar availability for growth.

The effect of warming could induce a complex interaction over the long term, such as affecting overall bud and stem development and/or promoting a selective pressure on adaptative capacity (Blumstein et al. 2024). Given the complexity of interactions among the internal processes, it is critical to understand potential adjustments of functional traits and sugar dynamics under higher temperatures to understand the impact on primary growth. Boreal species such as balsam fir or black spruce are between the species that could initially benefit from rising temperatures by increasing its photosynthetic capacity (Girardin et al. 2016). However, it has been shown that this effect may not be expected if there are also stressors such as dry periods, where the photosynthetic capacity could be lower than normal conditions due to the incapacity of the plants to maintain photosynthetic rates and prevent hydraulic failure (i.e. no warming or drought, Girardin et al. 2016; Reich et al. 2018). Leaf functional traits, such as leaf area (LA) and specific leaf area (SLA, i.e. a function dependent on leaf dry matter content and leaf thickness), provide a snapshot of plant performance, resource acquisition, and response to environmental stressors (Niinemets 2001; Mencuccini et al. 2019). For example, SLA is generally negatively related with temperature (Poorter et al. 2009); a small SLA implies a thicker leaf and more mass that prevent dehydration; however, this response in boreal ecosystems is nonlinear. In conifers, generally, there is an increase in SLA with crown depth; black spruce and balsam fir correspond this rule due to the optimization on nitrogen content per unit area (Larocque et al. 2014).

Defoliation is a biotic factor known to disrupt NSC production, allocation to growth, and phenology (Cooke et al. 2021). Starch allocation to storage decreases proportionally with increasing defoliation in both coniferous (Deslauriers et al. 2015; Fierravanti et al. 2019) and deciduous trees (Gross 1991; Kolb et al. 1992), indicating gradually lower C storage. The drastic decline in starch reserves is caused by a decrease in branch growth and LA, which is reduced by defoliation in the current or previous years (Gross 1991). LA is also directly affected by defoliation (i.e. when insects ingest whole or partial leaves) or indirectly, via reduced growth, thereby reducing the photosynthetic capacity of defoliated trees (Cooke et al. 2021). Interestingly, despite a decrease in starch reserves and total available sugars, an earlier bud opening is the most frequent response of defoliated trees (Kolb et al. 1992; Quiring and McKinnon 1999; Carroll and Quiring 2003; Deslauriers et al. 2019) The previous year carbon reserves being low with high structure maintenance cost, carbon could preferentially be allocated to bud opening and primary growth (Deslauriers et al. 2019) avoiding further tree decline.

Defoliation studies have also reported effects on functional traits, including an overproduction of leaves in *Quercus ilex* (Le Roncé et al. 2020), a decrease in preformed pairs of needles in *Pinus sylvestris* (Millard et al. 2001), and a decrease in bud mass in *Picea mariana* (Cartenì et al. 2023). Such changes potentially affect phenology. Indeed, functional traits influence phenology among both woody (Bolmgren and D. Cowan 2008; Horbach et al. 2023) and herbaceous (Valencia et al. 2016; Sporbert et al. 2022) species. Across functional groups of herbaceous species, a smaller SLA has been correlated to an earlier leaf and flower phenology (Valencia et al. 2016). A similar response has been observed for 19 species of deciduous trees (Horbach et al. 2023). Similarly, in broadleaved species, defoliation reduces LA at the expense of photosynthetic activity (Gottardini et al. 2020), which has consequences in the sugar-associated physiological processes. However, it remains uncertain as to how the shoot and leaf functional traits of boreal conifers under defoliation influence bud opening, as both source and sink could change.

**Figure 1.**
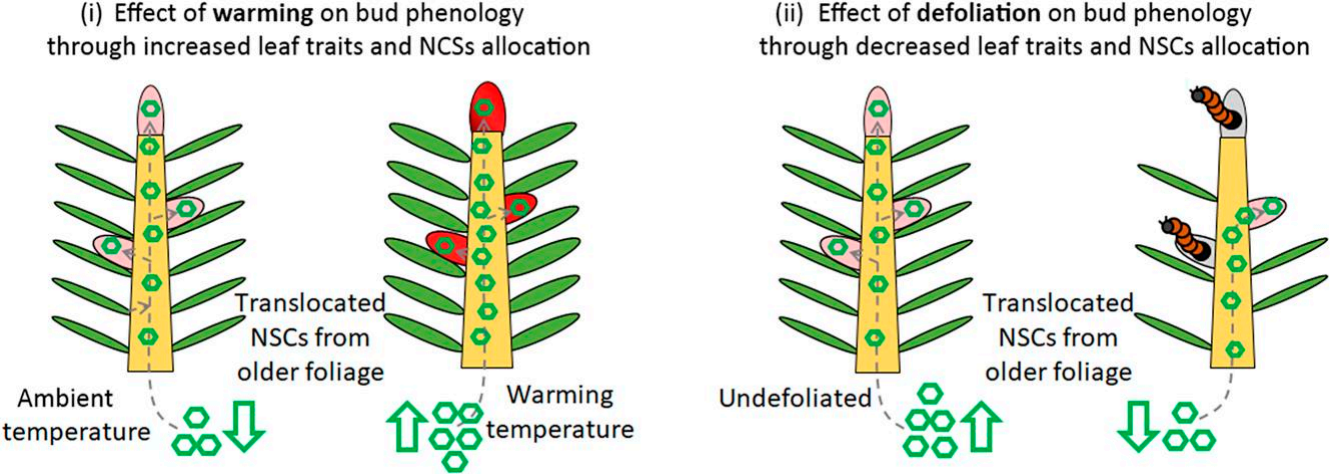
Representation of hypothesis for C-allocation in growing buds under either defoliation or warming. Shoot–needle allometric traits and NSC concentrations in needles, shoots, and buds (i) increase with heating and (ii) decrease with defoliation. Heating (i) in combination with defoliation (ii) affects phenology through changes in functional traits, via NSCs or both. Ambient temperature buds (in pink) and heating temperature buds (in red). The sugars are represented with green hexagons and translocation processes with gray arrows.

Here, we study 2 conifer species, balsam fir (*Abies balsamea* L. Mill) and black spruce [*P. mariana* B.S.P. (Mill.)], subjected to warming and defoliation, i.e. produced by the eastern spruce budworm (*Choristoneura fumiferana* Clemens), to assess how shoot–needle, i.e. apical and lateral branches, internodes and needles, functional traits, and the allocation of NSCs influence bud phenology in spring. We tested the hypotheses that shoot–needle allometric traits and NSC concentrations in needles, shoots, and buds (i) increase with warming and (ii) decrease with defoliation (Fig. 1). We also hypothesize that (iii) both warming and defoliation affect phenology through changes in functional traits or via NSCs (Fig. 1). We thus expect that both treatments will lead to an early bud break with (iv) warming, leading to wider leaf trait, and thus higher sugar allocation to primary growth and (v) defoliation, leading to a decreasing leaf traits and thus lower sugar allocation to primary growth.

## Results

### Bud phenology

**Figure 2.**
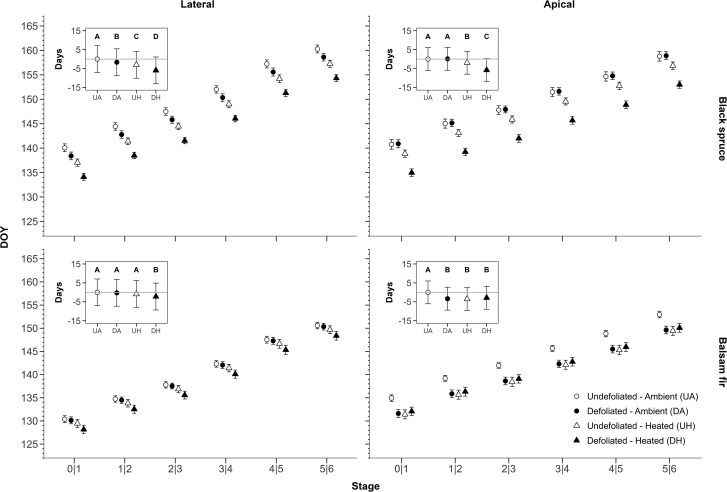
DOY at which there is a 50% probability of transitions to the following bud burst phase from Phase 1 to 6 for both lateral and apical buds in black spruce and balsam fir. The date of transition between phases was found with ordered probit regressions. The inset figure represents the mean difference, in days, between the control (undefoliated and ambient) and the other treatments. Different letters indicate significant differences between treatments (*P* < 0.05), illustrated by species. Sds are illustrated with vertical bars.

The transition of phenological phases for both lateral and apical buds was observed for each species and treatment (Fig. 2). In general, the bud-opening phases had a linear increasing trend over time [expressed in day of the year (DOY), time effect *P* < 0.0001, Table 1].

**Table 1.** Results of the ordinal logistic regression showing the effects of species, heating, and defoliation on the bud-opening phase, expressed as DOY, for both apical and lateral buds.

Source of variation	Apical bud			Lateral bud		
	Df	Chi-square	*P*	df	Chi-square	*P*
DOY	1	1313.38	**<0**.**0001**	1	2803.85	**<0**.**0001**
Species	1	269.85	**<0**.**0001**	1	919.28	**<0**.**0001**
Defoliation	1	26.92	**<0**.**0001**	1	54.23	**<0**.**0001**
Heating	1	65.91	**<0**.**0001**	1	149.10	**<0**.**0001**
Species × defoliation	1	0.56	0.4525	1	13.60	**<0**.**0001**
Species × heating	1	14.51	**<0**.**001**	1	28.32	**<0**.**0001**
Defoliation × heating	1	0.00	0.9725	1	7.71	**0**.**0055**
Species × defoliation × heating	1	37.87	**<0**.**0001**	1	0.08	0.7822
AIC	1098.81			2128.77		

Significant results are highlighted in bold.

For apical buds, the interaction of species × defoliation × heating was significant (*P* < 0.0001, Table 1). For spruce grown at an ambient temperature, the transition from Phase 5 to 6 occurred at DOY 159. Under heating conditions, both undefoliated and defoliated trees had an earlier bud-opening phase, occurring 2 and 6 d earlier, respectively, than the nonheated trees (Fig. 2, inset graph). In fir, the phenology of undefoliated–ambient (Stage 6) trees occurred significantly later, at DOY 152.5 (Fig. 2, inset graph). The phenology was earlier for all other treatments (Fig. 2, inset), with bud opening 3 to 3.5 d earlier in both the heated and defoliated trees. Species was also highly significant (*P* < 0.0001; Table 1), with fir having an earlier phenology than spruce, with a mean difference of about 6 d between species.

Lateral buds showed a similar trend over time as apical buds, although there were some differences among the treatments. Both the species × defoliation and species × heating interactions were significant (*P* < 0.0001, Table 1). Indeed, fir phenology was significantly earlier (approx. 3 d) but only for heating × defoliation trees. In comparison, defoliation, heating, and their combination all had an earlier bud-opening phase for spruce with approx. 6 d difference between ambient trees and heating × defoliated trees.

### Needle and shoot allometry

Positive correlations were found between allometric traits, most being significant (Table 2). Leaf traits, including new growing internode number of needles (Nb_new_), LA (LA_new_), and SLA (SLA_new_), were mostly positively correlated (Pearson *r* = 0.28 to 0.83). LA_new_ also correlated positively with the volume of the new (V_new_) and old internodes (V_old_). The SLA of older internodes (2017 to 2018) correlate with all leaf traits, (*r* = 0.293 to 0.83) except with volume (Table 2). Because LA was correlated and was similar to SLA, it was removed from the MANOVA test.

**Table 2.** Correlation matrix (Pearson correlation, *r*) of allometric traits, phenological Stage 6, and duration of bud opening

	End	D	Nb_new_	Nb_old_	LA_new_	LA_old_	SLA_new_	SLA_old_	V_new_	V_old_
Onset	**0**.735***	*−0.067^NS^*	0.225^NS^	**0**.289*	*−0.149^NS^*	*−0.219^NS^*	*−0.096^NS^*	*0.146^NS^*	*0.003^NS^*	−**0**.369**
End		0.627***	0.206^NS^	**0**.416**	*−0.230^NS^*	*−0.176^NS^*	*−0.179^NS^*	*0.052^NS^*	*−0.005^NS^*	−0.471***
D			0.041^NS^	**0**.280*	*−0.161^NS^*	*−0.007^NS^*	*−0.148^NS^*	*−0.092^NS^*	*−0.011^NS^*	*−0.269^NS^*
Nb_new_				**0**.552***	0.832***	0.495***	0.287*	0.392**	0.343**	0.222^NS^
Nb_old_					0.289*	0.734***	0.237^NS^	0.418**	0.055^NS^	0.069^NS^
LA_new_						0.629***	0.334*	0.293*	0.430**	0.544***
LA_old_							0.542**	0.417***	0.229^NS^	0.511***
SLA_new_								0.836***	*−0.022^NS^*	0.088^NS^
SLA_old_									*−0.027^NS^*	*−0.038^NS^*
V_new_										0.478**

Significant correlations are highlighted in bold with negative correlations are italized. All trees (*N* = 48) were included in the correlation, irrespective of species and treatment.

NS, nonsignificant correlation (*P* > 0.05); significant correlations are indicated by asterisks, *(*P* < 0.05), **(*P* < 0.01), and ***(*P* < 0.001). Legend for phenology and allometric traits: onset, beginning of bud-opening Phase 1; end, end of bud-opening Phase 6; D, duration of bud opening; Nb_new_–Nb_old_, number of needles (*n*) in the new 2019 and older internode (2017 to 2018); LA_new_–LA_old_; LA (m^2^); SLA_new_–SLA_old_, SLA (m^2^·kg^−1^); V_new_–V_old_, internode wood volume (cm^3^).

**Figure 3.**
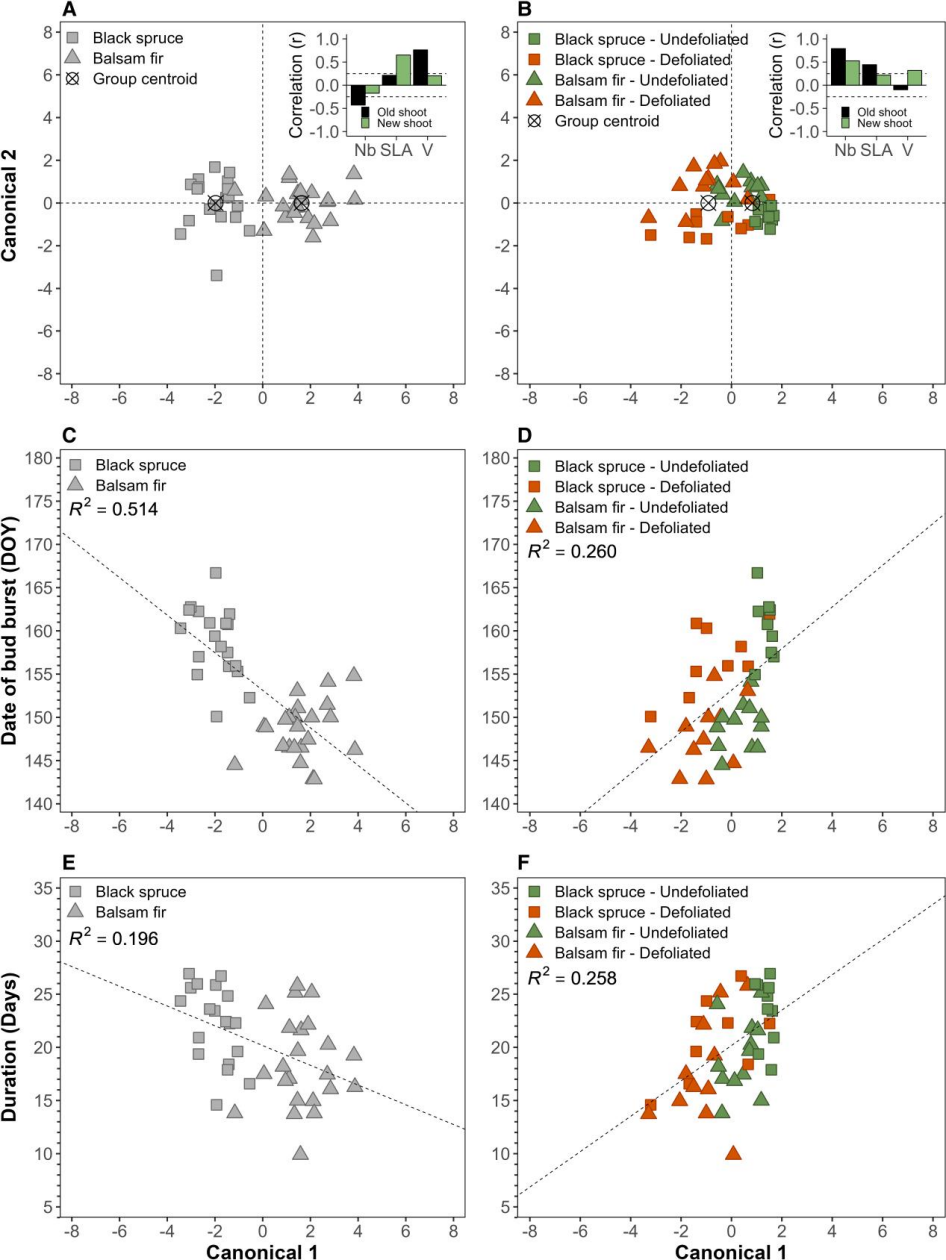
Relationships between bud phenology and needle and shoot allometry. Top panels **A** and **B)**: canonical discriminant analysis using a combination of allometric traits, including internode wood volume (V_new_ and V_old_, cm^3^), number of needles (Nb_new_, Nb_old_), and SLA (SLA_new_, SLA_old_, m^2^·kg^−1^) to illustrate the **A)** species and **B)** defoliation effect (*N* = 48). The insets **A** and **B)** represent Pearson correlations (*r*; *P* < 0.05) between the allometric traits and Can1. Correlations of *r* < 0.25 are nonsignificant (*P* > 0.05). The correlations for older internodes are illustrated in black, whereas the newly formed internodes are in green. Bottom panels **C** to **F)**: linear regression (*P* < 0.05) between Can1 and date of bud burst considering the **C)** species effect and **D)** defoliation (*N* = 48). Regression (*P* < 0.05) between Can1 and the duration of bud burst considering the **E)** species effect and **F)** defoliation (*N* = 48).

The allometric traits of shoots and needles (Table 3, Fig. 3, and Supplementary Fig. S1) differed between species (*P* < 0.0001) and defoliation (*P* = 0.0009). In contrast, heating the other factor combinations did not influence the allometric traits with *P* > 0.05 (Table 3). In regard to species (Fig. 3A), the canonical axis 1 (Can1) divided fir (positive scores) and spruce (negative scores). Can1 was positively correlated with SLA_new_ (*r* = 0.65) and V_old_ (*r* = 0.76) (Fig. 3A, inset); higher values of these traits were found for fir. In contrast, Can1 was negatively correlated with Nb_old_ (*r* = –0.43); thus, spruce had a higher number of old needles than fir.

**Table 3.** MANOVA testing the effect of species, defoliation, and heating on allometric traits (number of trees used, *N* = 48)

Factors	Wilds' λ value	*P*
Species	**0**.**1610**	**<0**.**0001**
Heating	0.8911	0.8523
Species × heating	0.5829	0.0549
Defoliation	**0**.**3670**	**0**.**0009**
Species × defoliation	0.7051	0.2384
Heating × defoliation	0.8383	0.6709
Species × heating × defoliation	0.8986	0.8687

Significant effects are highlighted in bold.

Can1 discriminated most of needle traits to separate undefoliated (positive score) from defoliated (negative score) trees (Fig. 3B and inset). Given the correlation between the traits and Can1, undefoliated trees were generally characterized by a higher number of needles and greater SLA in both the older shoots (2018 to 2019) and the new 2019 shoots and the converse for defoliated trees (Fig. 3B and inset and Supplementary Fig. S1). Higher V_new_ (*r* = 0.31) also characterized undefoliated sapling.

### Relationships between bud phenology and needle and shoot allometry

Both the onset (phenological Stage 1, open bud) and end (Stage 6, exposed shoots) of bud phenology were highly correlated (*r* = 0.735; Table 2), whereas only the end was correlated with duration (D; *r* = 0.627). The end, considered as the date of bud burst, correlated positively with Nb_old_ (*r* = 0.289), indicating that earlier phenology corresponds to reduced needle number. A negative correlation with the volume of the old internodes was found for both the onset (*r* = −0.369) and end (*r* = −0.471) of bud phenology, indicating that larger internodes lead to an earlier phenology. The duration of phenology [from Stage 1 (onset) to Stage 6] has only a weak correlation with the number of needles in the old internodes (Table 2).

The end of bud phenology (Stage 6, considered as bud burst) was then linked to Can1 (linear regression) to test the influence of the combination of allometric traits for both species (*R*^2^ = 0.51; *P* < 0.0001; Fig. 3C) and defoliation (*R*^2^ = 0.26; *P* < 0.0006; Fig. 3D). Similar results were found for the duration of bud opening (Fig. 3, E and F), although with a lower *R*^2^ for species. In regard to the species effect, an earlier and shorter phenology (mostly observed in fir) was characterized by larger allometric traits, mostly V_old_ and SLA_new_. For the defoliation effect, an opposite trend to species was observed, showing smaller leaf traits corresponding to an earlier phenology. Indeed, an earlier phenology (negative scores mostly representing defoliated trees; Fig. 3B) was characterized by smaller allometric traits, thus a lower Nb_new_, Nb_old_, SLA_old_, and V_new_.

### Sugar variation among organs, species, and treatments in relation to phenological stage

**Figure 4.**
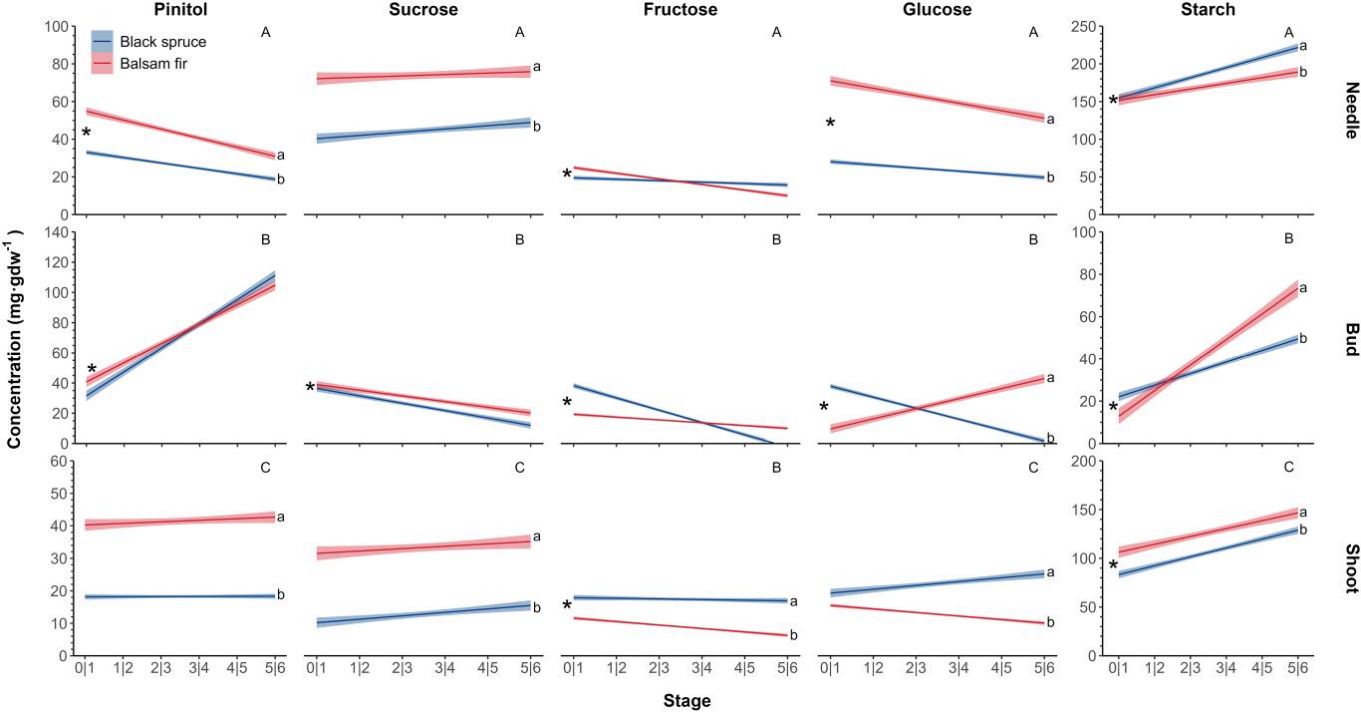
Predicted NSC concentrations (mg·g dw^−1^) during bud-opening phases in needles, buds, and shoots. The NSC includes glucose, fructose, pinitol, sucrose, and starch for black spruce (in blue) and balsam fir (in red). The shaded areas correspond to the 95% CIs. Significant slopes representing the effect of phase (*P* < 0.05) are identified with an asterisk, and significant differences between species are identified with lowercase letters. Capital letters indicate significant differences (*P* < 0.05) among organs for a given sugar. All results were generated by the generalized linear mixed model analysis using the prediction of the model.

**Figure 5.**
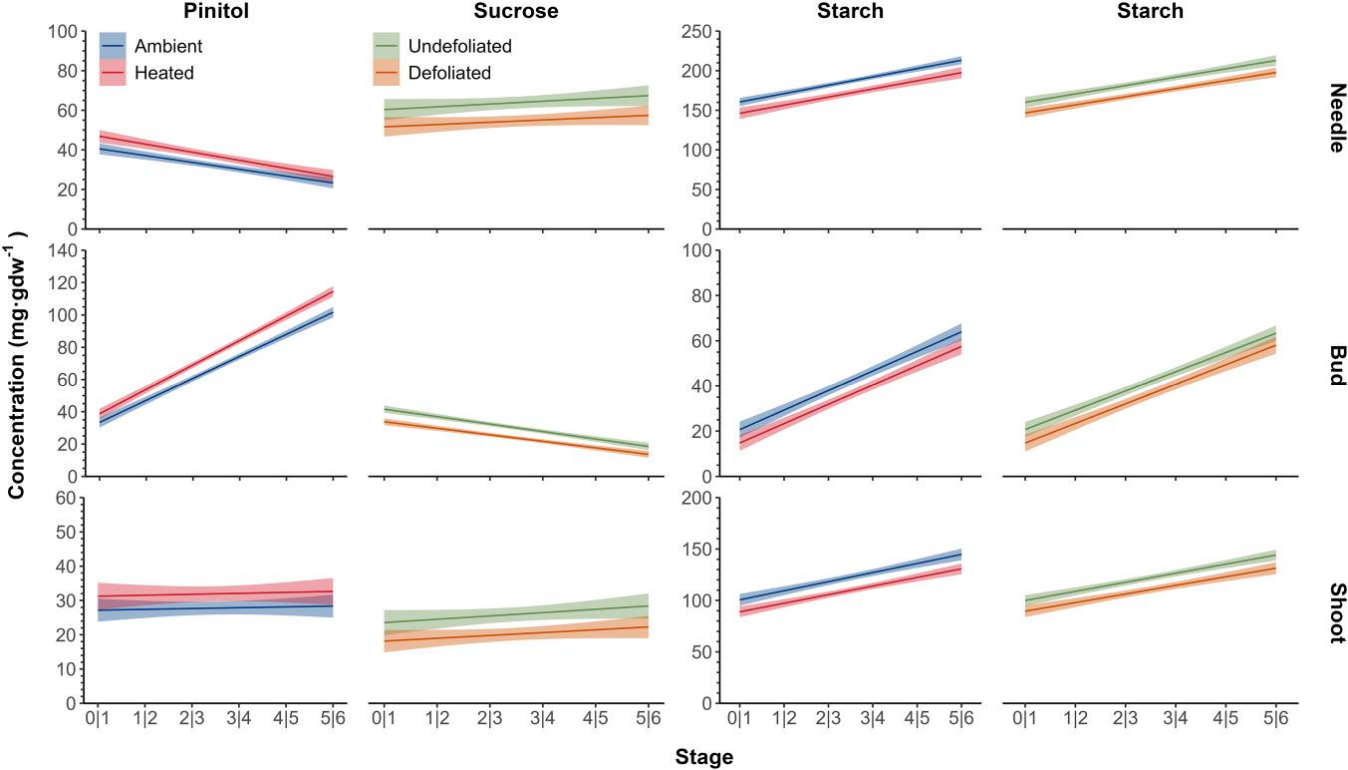
Predicted NSC concentrations (mg·g dw^−1^) for bud-opening phases in needles, buds, and shoots, illustrating the effect of heating (for pinitol and starch) and defoliation (for sucrose and starch). The shaded areas correspond to the 95% CIs. All results were generated by the generalized linear mixed model analysis using the prediction of the model.

The sugar concentration during spring was illustrated for each species, treatment, and organ (Supplementary Figs. S2 and S3). Along phenological stages, significant differences were found that were compound—and organ—specific (Table 4 and Figs. 4 and 5).

**Table 4.** Results of the generalized linear mixed model analysis showing the effects of bud burst stage, organ, species, heating, and defoliation on the concentration of fructose, glucose, D-pinitol, and sucrose.

Effect	Fixed effects tested	Sugars				
		D-pinitol	Sucrose	Fructose	Glucose	Starch
		*P*	*P*	*P*	*P*	*P*
Phenological stages across organ and species	Stage	**<0**.**0001**	**<0**.**0001**	**<0**.**0001**	**<0**.**0001**	**<0**.**0001**
	Organ	**<0**.**0001**	**<0**.**0001**	**<0**.**0001**	**<0**.**0001**	**<0**.**0001**
	Organ × stage	**<0**.**0001**	**<0**.**0001**	**<0**.**0001**	**<0**.**0001**	**<0**.**0001**
	Species	**<0**.**0001**	**<0**.**0001**	**0**.**0025**	**<0**.**0001**	0.2360
	Species × stage	0.0560	0.3019	**0**.**0007**	**<0**.**0001**	**0**.**0040**
	Species × organ	**<0**.**0001**	**<0**.**0001**	**<0**.**0001**	**<0**.**0001**	**<0**.**0001**
	Species × stage × organ	0.2393	0.0969	**<0**.**0001**	**<0**.**0001**	**<0**.**0001**
Heating and defoliation across species	Heating	**0**.**0086**	0.8463	0.1266	0.1952	**0**.**0068**
	Defoliation	0.1943	**0**.**0036**	0.7511	0.6213	**0**.**0119**
	Heating × defoliation	0.4549	0.6873	0.5995	0.9044	0.9632
	Species × heating × defoliation	0.6888	0.4960	0.4330	0.0662	0.2888
Fit statistics						
−2log		−812.4	2573.8	−393.5	−167.5	2697
AIC		−806.4	2581.8	−389.5	−161.5	2705

Significant results were highlighted in bold.

The organ-specific pattern of sucrose along phenological stages differed through the various bud break stages. Sucrose showed no stage effect in needles and twigs, whereas it decreased through the stages in buds, explaining the organ × stage (*P* < 0.001) effect. In buds, sucrose decreased through Stage 1 to 6 by approximately 22 and 17 mg·g dw^−1^ less sucrose in spruce and fir, respectively, with no effect between species. Sucrose concentrations were higher in fir than in spruce in needle and stem, explaining the species × organ effect (*P* < 0.0001), differing by 21 and 30 mg·g dw^−1^, for twigs and needles, respectively, between fir and spruce. Defoliation reduced the concentration of sucrose (*P* = 0.0036), with a reduction in needles of 10 mg·g dw^−1^less in defoliated and 5 to 7 mg·g dw^−1^less in buds and twigs.

**Figure 6.**
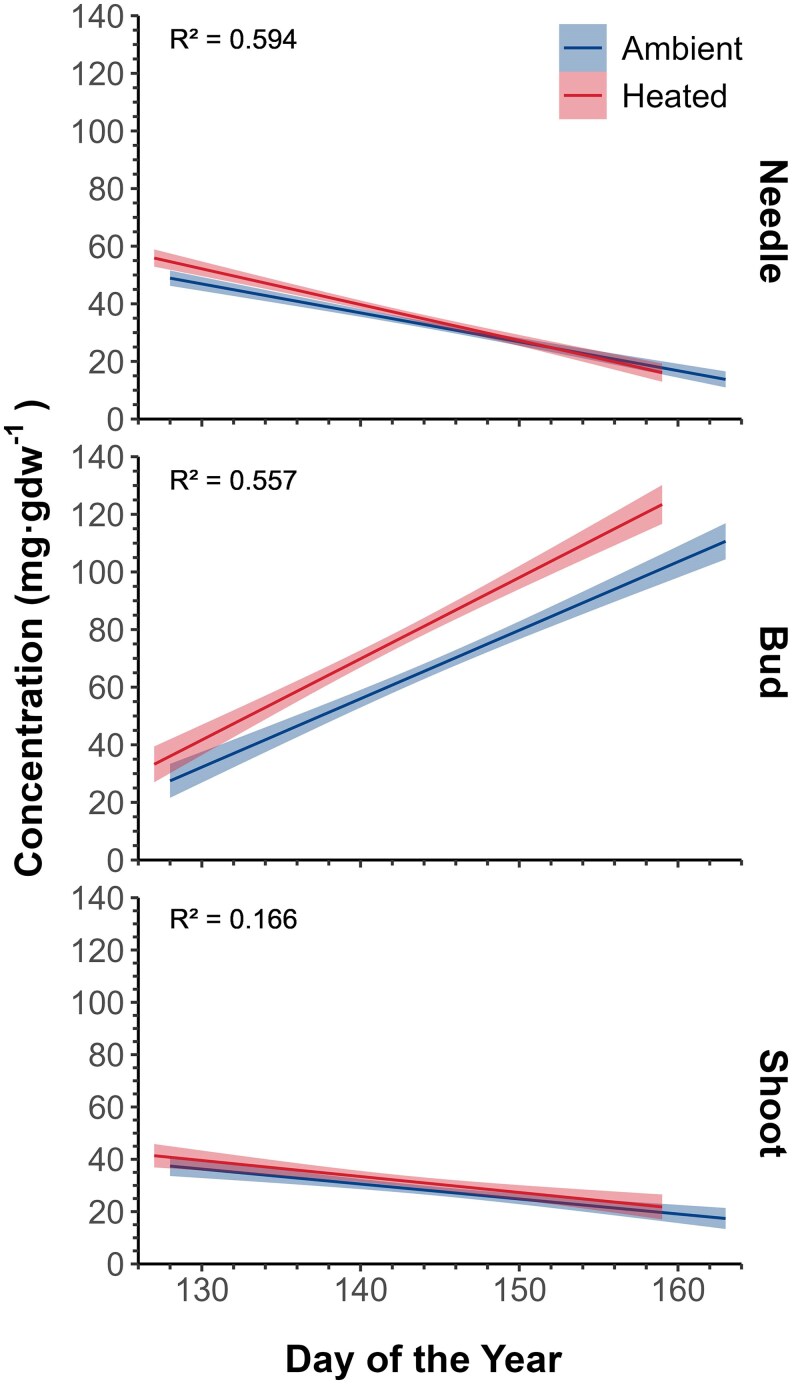
Predicted pinitol concentrations (mg·g dw^−1^) in relation to time (expressed in DOY) through all bud burst phases (from 1 to 6). Linear regressions (*P* < 0.05) were performed and illustrated for ambient (blue) and heated (red) temperatures for needles, buds, and shoots. The shaded areas correspond to the 95% CIs.

The observed trend for pinitol concentrations through the phenological stages depended on the organ (organ × stage, *P* < 0.001, Table 4). Pinitol increased in buds through the phenological stages while decreasing in needles and remaining more or less constant in twigs (Fig. 4). Pinitol concentrations were higher in fir, except in buds (species × organ effect; *P* < 0.0001). However, pinitol concentration had a similar trend between species across phase (species × phase effect; *P* = 0.0560). In buds, pinitol increased from 40 to 110 mg·g dw^−1^ between Stages 1 and 6, with similar values between species. Heating had a significant effect on pinitol concentration (*P* = 0.0086), which was higher in the heated than ambient trees (Table 4 and Fig. 5). Depending on the phenological phase, pinitol increased about 7 to 14 mg·g dw^−1^ under warming in buds relative to the ambient treatment. In needles and shoots, pinitol increased approx. 3 to 5 mg·g dw^−1^ under warming. This pattern was confirmed by relating pinitol and the DOY at which each phase occurred. Heated plants, which began phases at an earlier DOY, had higher pinitol concentrations (Fig. 6). The difference was particularly marked in buds.

In all organs, the hexoses (glucose and fructose) mostly decreased along the bud-opening phases with different trends according to species, explaining the species × phase × organ effect (*P* < 0.0001; Table 4) as well as the other cross effects. The decrease in fructose through the phenological phases was significant for all organs (*P* < 0.0001). The amount of fructose was similar between species in needles and buds, although higher in fir for shoots, explaining the significant interaction species × organ. However, fir had much higher glucose concentrations in the needles (∼37 mg·g dw^−1^) with a species × organ effect (*P* < 0.0001). For both hexoses, lower concentrations were found in the shoots than in needles and buds (Fig. 4).

Starch concentration increased in all organs through the phenological stages with a greater increase in needles and buds, depending on the species (interaction species × stage × organ; *P* < 0.0001) (Fig. 4). In needles, the concentration increased from 150 to 185 to 230 mg·g dw^−1^, with a higher concentration in spruce, increasing with stages. Similarly, the concentration in buds increased from 20 to 50 to 85 mg·g dw^−1^, with a higher concentration in fir at the latest stages. Both defoliation (*P* = 0.0119) and heating (*P* = 0.0068) reduced starch concentrations (Fig. 5). Under warming, the reduction in needles was about 17 to 20 mg·g dw^−1^ lower than the control, while in defoliation, starch was reduced by about 15 mg·g dw^−1^ (lower than the nondefoliated). In buds and stem, reduction between 10 and 15 mg·g dw^−1^ was observed for both heating and defoliation treatments. However, no defoliation × heating and species × defoliation × heating interactions was detected, confirming a similar effect for both treatments and species.

## Discussion

### Influence of warming and defoliation on bud burst

As expected, fir opened its buds earlier than spruce, with a mean difference of 6 d, confirming the species' life history under natural conditions (Antonucci et al. 2015; Rossi and Isabel 2017). The phenology of the 2 species diverges even more at a larger scale, e.g. across Canada, where the differences are up to 17 d (Podadera et al. (2024). The response of phenology to the combination of heating and defoliation related to species and type of bud (apical versus lateral). In fir, apical buds under either heating or defoliation were associated with an earlier bud burst of 3 to 3.5 d. However, lateral buds responded only to the combination heating × defoliation, with an earlier emergence of 3 d.

When the seedlings were warmed, the observed difference was similar to those reported by Bellemin-Noël et al. (2021), with an earlier phenology of about 2 d in fir and spruce. Both apical and lateral buds of spruce were highly responsive to heating or heating in combination with defoliation, about 6 d earlier than the control (undefoliated and ambient temperature). The phenology of northern conifers, such as spruce, is highly sensitive to temperature (Mura et al. 2022; Gao et al. 2023). For spruce, a rate of 5.4 d per degree Celsius was reported for the SPRUCE experiment in Minnesota, United States (Cartenì et al. 2023).

For both apical and lateral buds of both taxa, defoliation advanced spring phenology, confirming previous findings for the same species (Deslauriers et al. 2019; Ren et al. 2020). However, the difference in days (2 to 6 d earlier depending on buds, apical versus lateral) was lower in this field setting than in a controlled environment (growth chamber or greenhouse), especially for lateral buds, probably because of the effect of other environmental conditions (Deslauriers et al. 2019; Ren et al. 2020).

### Contrasting influence of functional leaf traits on phenology

Species with greater allometric traits, such as SLA and wood volume, have an earlier phenology, reflecting the strategy of resource acquisition for growth. Meanwhile, the heating treatment did not have a significant effect in the species phenology; an earlier phenology was related to a lower SLA and number of needles because of defoliation, especially in spruce. These differences under defoliation probably reflected a reduced demand or competition for growth, as discussed in the next paragraphs.

Fir, which has the earliest reactivation, was characterized by a greater SLA_new_ and V_old_. Allometric traits are good indicators of plant functioning and photosynthetic capacity (Wright et al. 2004), with leaf and stem hydraulic traits being somewhat linked together (Song et al. 2022). Wood volume is related to water storage and transport and mechanical stability (Taneda and Tateno 2004), whereas SLA reflects carbon resource acquisition and growth rate (Song et al. 2022). Therefore, a higher leaf SLA may require a greater shoot volume to support the water supply for CO_2_ assimilation. In fir, a greater SLA and biomass explain the higher NSC concentrations (sucrose, pinitol, and glucose) found in the needles due to its relationship with a higher productivity related to those traits (Dumschott et al. 2017). However, NSC concentrations in growing buds (i.e. metabolite pool for soluble sugars) remain similar to those of spruce, even with a lower SLA. In addition to potential genetic differences, the species allometry reflects resource acquisition in terms of carbon and water for bud swelling and their phenology.

Under defoliation, a lower number of existing (Nb_old_) and growing (Nb_new_) needles were associated with an earlier bud burst. In conifers, the predetermined size and number of embryonal organs in the bud influence current shoot growth (Owens et al. 1977; Lipavská et al. 2000) and the carbon partitioning among buds (i.e. sink competition). As defoliated trees had smaller leaf traits, the earlier bud opening could thus be explained by a reduced growth sink (in terms of number or mass), given the drop in buds and new internodes’ needle and volume. Under defoliation conditions, buds and new foliage are eaten while they are growing (Supplementary Fig. S5), thus removing important sinks (partially or entirely) and decreasing all leaf traits. According to Cartenì et al. (2023), this has the effect of allocating carbon resources over fewer growing sinks, even if a reduction in sucrose is observed in needles and buds.

### Temporal dynamics of sugar and starch allocation in needles, shoots, and buds during bud opening

In each analyzed organ (buds, needles, and shoots), NSC concentrations varied through the various phenological phases, illustrating specific metabolic processes, leading to growth reactivation (Cooke et al. 2012; Singh et al. 2017). Energy to grow new shoots from buds in conifer originates from both reserves (i.e. storage starch) and the current photosynthesis of old needles (Gordon and Larson 1968; Little 1970). In spring, the warming temperatures induce the recovery of photosynthesis (Yang et al. 2020) and the conversion of sugars to starch during dehardening, leading to an increase in starch through the phenological stages (Schadel et al. 2009; Deslauriers et al. 2019; Schoonmaker et al. 2021). The increase in starch concentrations was greatest in needles, where starch accumulates in the chloroplast before the onset of shoot growth and acts as an inert carbon in the absence of a growth sink (MacNeill et al. 2017). Because of the elevated starch synthase enzymatic activity in chloroplasts (Crumpton-Taylor et al. 2013), hexose decreases in needles. In needles of *P. sylvestris* from cold sites, glucose and fructose, but not sucrose, have been shown to decrease during spring (from May to June) (Sleptsov et al. 2023), a finding that corresponds to our results. For sucrose, the nonsignificant trend across phenological phases reflects a constant concentration in needles, and thus the sugar partitioning between the hexose phosphate pool involves either starch synthesis (in chloroplasts) or sucrose synthesis (for phloem transport). The size of the mobile pool of sugars normally reflects the balance between the uptake and the demand for growth and respiration with probably a very limited variability.

Pinitol concentrations in needles dropped by half through the phenological phases (from 60 to 30 mg·g dw^−1^ in fir and from 35 to 20 mg·g dw^−1^ in spruce), as observed in *P. sylvestris* between May and June (Sleptsov et al. 2023). Dominguez and Niittylä (2022) suggest that pinitol synthesized in the leaf can be translocated into the phloem sap, thus representing a form of C transport in gymnosperms. This process likely explains the high pinitol concentrations found in the stem (representing bark, phloem, and xylem), which was slightly higher than that of sucrose (with 6 to 7 mg·g dw^−1^ more pinitol in the shoots). Therefore, such a lower concentration in needles may represent the translocation of pinitol in the stem, confirming the observations made for other species (Popp et al. 1997; Dumschott et al. 2017; Lahuta et al. 2018), and the maintenance of the buds as important sinks, with increasing concentrations of pinitol.

In the buds of both species, pinitol, sucrose, and starch showed the highest variability through the phenological phases, with an increase in pinitol and starch and a decrease in sucrose. In buds, the increase in starch was less than in needles but remained somewhat high for an active growing sink. Developing leaves possess starch granules composed of amylose and amylopectin, depending on their development stages (Merida and Fettke 2021), to sustain metabolism during the night (MacNeill et al. 2017). These starch granules are necessary for normal leaf development (Crumpton-Taylor et al. 2013). After swelling, first buds become translucent (Phase 4) and then shoots and needles are exposed (Phases 5 and 6), where green parts probably undergo photosynthesis while importing carbon from older needles (Pantin et al. 2012).

Despite the increasing photosynthetic activity of the tree, sucrose decreased during the new growing shoot during bud opening. This decrease is related to its catabolism, i.e. the combination of sucrose synthase and cellulose synthase used to build new leaf structures (Bonhomme et al. (2010). A different behavior was observed for pinitol in buds, which increased throughout the phenological phases. Moreover, the concentration of pinitol was similar between species. In buds, pinitol represents 44% of all carbohydrates in *Pinus cembra* (Hoch et al. 2002) and *Picea abies* (Lipavská et al. 2000). Pinitol concentration is also high in other growing sinks, such as the cambium zone during wood formation in conifers (Simard et al. 2013; Deslauriers et al. 2014).

### Higher pinitol concentrations under warming might accelerate bud break

As the measured allometric traits (number of needles, SLA, and internode volume) did not differ between the control and warmed trees, the advanced phenology under +2 °C warming could be metabolically related to increased pinitol concentrations. D-Pinitol, a cyclitol synthesized from glucose-6-phosphate as the precursor via myo-inositol and D-ononitol (Sánchez-Hidalgo et al. 2021), is considered more stable than other carbohydrates, i.e. not readily metabolized. This confers to pinitol the ability to rapidly accumulate, working as a carbon sink (Merchant and Richter 2011), or a pool of osmolyte accumulation (acting as storage; Supplementary Fig. S4), leading to osmotic adjustment (Turner 2018). Transporters were reported to actively import sugar alcohol in the plasmalemma or in the tonoplast membranes (Zhou et al. 2022), therefore contributing to osmotic and turgor pressure. Indeed, the increasing concentrations of pinitol across phenological stages (+170%) and under warming (+17%) enhance the solute content (i.e. osmolarity), therefore decreasing the osmotic potential (ψ_s_). The decrease in ψ_s_, mediated by sugar alcohols, can maintain or strengthen water absorption (Dumschott et al. 2017) and thus turgor pressure (ψ_p_) under growth (Pantin et al. 2012; Zhang et al. 2024). It may also help maintaining growth under water deficit (Turner 2018), which can occur in warmer conditions. At the cellular level, reduced and somewhat inert carbon sink, such as pinitol, could also prevent photosynthesis down-regulation in the growing buds (Merchant and Richter 2011). Even if the influence of sugar alcohols on growth is still unknown (Dumschott et al. 2017), pinitol could play many roles in the growing needles and might accelerate swelling under warming.

On the other hand, higher temperatures lead to lower starch reserves; therefore, less starch was accumulated under a 2 °C warming. Warming is often associated with higher metabolic rates (Dusenge et al. 2019), consuming C during respiration and thereby reducing starch reserves. Under warming conditions, a lower replenishment in starch reserves has been observed in spruce (Balducci et al. 2015), matching our results. No other sugar was affected by warming, in contrast with previous studies of the same species studying the effects of warming on hexose (Way and Sage 2008).

### Decreased sucrose and starch under defoliation does not delay phenology

Defoliation reduced sucrose concentrations in all organs, with a drop of 30% in buds. In a complete defoliation experiment, Li et al. (2002) observed 53% less soluble sugars in the current-year needles, a level not attained in our experiment. A lower concentration of soluble sugars was also observed in defoliated fir and spruce at the beginning of the summer (June to July) (Fierravanti et al. 2019), matching our results. Starch concentrations in buds were generally 15% lower under the defoliation conditions. Defoliation reduces both source and sink activities: source activity can be reduced by the formation of smaller leaves (e.g. area) and sink capacity because of the damage of the new growing shoot. Because starch accumulation depends on current photosynthesis, the spring pool is greatly reduced with needle loss (Vanderklein and Reich 1999). Starch reserves in spring thus decrease when conifers are subjected to successive years of defoliation (Deslauriers et al. 2019).

## Conclusions

Both black spruce and balsam fir had an earlier phenology under warming and defoliation conditions. However, different physiological mechanisms can explain this response. On the one hand, leaf functional traits and the growing shoot were smaller under defoliation, therefore reducing the sink demand and confirming that plants having a smaller growth unit experience earlier phenology (Fournier et al. 2020), even with a reduced NSC allocation to the growing unit. On the other hand, both older and current shoot and needle traits (i.e. the bud sink) remained similar under warming. However, pinitol production and its allocation in buds increased (i.e. the source effect) and could metabolically explain the earlier bud opening under warming. The production pathway and transport of pinitol should be studied under warming conditions to confirm these findings.

## Materials and methods

### Experimental design

The experimental site was located at the Valcartier Forest Research Station (46°56′N 71°29′W, 207 m a.s.l.), Quebec, Canada. The regional annual temperature is 3.4 °C, with a total precipitation of 1250 mm. Snow cover lasts generally from September to May and reaches a maximum depth of 136 cm.

Our study was conducted as a common garden experiment designed with triangle-like plots equipped with a metal frame surrounding the trees and sustaining a heating system with 6 1000 W infrared ceramic radiators placed 1.2 m from the ground (Supplementary Fig. S5) (T-FACE, temperature-free-air controlled enhancement system) (Bellemin-Noël et al. 2021). Six T-FACE plots were selected, 3 nonheated (control) and 3 heated, to increase the temperature by 2 °C from the end of April until the beginning of September. The control plots remained at ambient temperature but were also equipped with nonheating radiators to replicate any disturbance to the tree. Air temperature was monitored by APOGEE SI-11 sensors (Campbell Scientific Corporation, Logan, UT). In 2016, 3-yr-old balsam fir and black spruce saplings were planted. The fir and spruce saplings averaged 40 and 37 cm in height, respectively (Bellemin-Noël et al. 2021). These black spruce and balsam fir saplings originated from 2 adjacent ecological regions, forming 2 provenances (Saucier et al. 2011). However, giving the proximity, this effect was not considered in the analysis. Each plot was surrounded by 2 rows of seedlings to avoid edge effects.

In each plot, 8 trees were randomly selected for this study for a total of 48 individuals (4 trees × 2 species × 6 plots). Defoliation was naturally induced on half of the trees by implanting L2 stage eastern spruce budworm (*C. fumiferana* Clemens) instars (Deslauriers et al. 2019). The larvae came from the Insect Production Services laboratory at the Great Lakes Forestry Centre [code standard Glfc:IPQL:Cfum01 to Cfum16 (Roe et al. 2018)]. At the end of May 2018, the instars were randomly deposited on the buds and on the last whorl in groups of 10 to 12, for a total of 100 to 120 larvae per tree. A white fine tulle net (Proteknet 60 g, mesh size of 1.9 × 0.95 mm; light transparency at 93%) covered each tree to protect the surrounding environment (Supplementary Fig. S5), including the control trees. In May 2019, additional instars were added to ensure an efficient defoliation effect, i.e. from moderate (>50% of new shoot defoliation) to severe (>75% of new shoot defoliation) defoliation. Therefore, the saplings cumulated 2 years of defoliation on new growing shoot (internodes formed in 2018 and 2019; Supplementary Fig. S1). The damages were reported in the leaf's traits of 2 sampling branches (see allometry measurements), but defoliation induced by the budworm was evenly distributed on the tree.

### Phenology and allometry

During 2019, bud burst timing was monitored on the trees 3 times per week from DOY 128 to 168. Observations of the phases of 2 lateral branches of each tree were performed according to Rossi and Isabel (2017) by using a scale from 0 to 6, where Phase 0 represents a dormant bud; Phase 1, an open bud; Phase 2, an elongated bud; Phase 3, a swollen bud; Phase 4, a translucent bud; Phase 5, a split bud; and Phase 6, an exposed shoot.

In the autumn of the same year, we collected 2 intact lateral branches per tree (2 branches × 48 trees) and stored them in plastic bags before freeze-drying the samples for 5 d. The old (i.e. produced in 2017 to 2018) and the new (i.e. produced in 2019) internodes of the branches were considered separately for the analysis of shoot and needle traits. The needles were detached from the internodes. All needles were counted and measured with a digital scanner at 800 dpi using the software WinSEEDLE version 2019a (Regent Instruments Inc., Quebec, QC, Canada) to obtain the projected LA (cm^2^) and their number (Nb). The needles' dry mass (g) was weighed after oven-drying at 65 °C for 48 h (Morabito et al. 2006). The SLA (m^2^·kg^−1^) was calculated as the LA ratio to leaf mass. The length and diameter (mm) of the internodes were measured with an electronic caliper and used to estimate the volume (V, cm^3^) of an approximate truncated cone. The volume of the 2017 and 2018 internodes was summed to determine old wood volume (V_old_), whereas the sum of the 2019 internodes gave the new wood volume (V_new_) (Supplementary Fig. S5).

### NSCs

Analyses of NSCs were carried out weekly in the spring 2019, from the end of April, during dehardening (April 27), to the end of bud burst (June 12). At each sampling date, a shoot section was cut from each tree and frozen at −20 °C. Buds, twigs, and needles were separated (Supplementary Fig. S5), immersed in liquid nitrogen to stop metabolic activity, and then freeze-dried for 5 d. Afterward, samples were reduced to a powder using a ball mill (Mixer Mill MM 400, RETSCH).

The sugars were extracted from plant tissues using a method adapted from Lunn et al. (2006). A 10 mg subsample was weighed in a microcentrifuge tube. Samples were dissolved in 500 *µ*L of a CHCl_3_/CH_3_OH mixture (3:7), vortexed for 5 min, and incubated for 2 h at −20 °C. After incubation, 200 *µ*L of 0.5 g/L D-glucose-^13^C_6_ in MilliQ water was added as an internal standard. The tubes were kept cold on an ice bath between each step of the extraction process. After shaking, tubes were centrifuged at 1400 rpm for 10 min at room temperature. The supernatant was transferred to a microcentrifuge tube, and the sugars were then extracted a second time by adding 200 *µ*L of cold water and shaking for 1 min. The supernatant obtained after centrifugation for 10 min at room temperature was combined with the first. The aqueous extracts were evaporated in a vacuum concentrator at 35 °C and 1725 rpm for 3 h. The sample was reconstituted in 1000 *µ*L of acetonitrile/water 9:1 and then diluted to a 1/10 ratio using the same solvent mixture. Before analysis with LCMS, the extracts were filtered at 0.45 *µ*m.

LCMS analyses were performed using an Agilent 6546 ESI-Q-TOF mass spectrometer coupled to an Agilent 1260 Infinity II LC system equipped with a Luna Omega Sugar column (Phenomenex, 2.1 × 100 mm, 3 *µ*m particle size). The LC parameters were an injection volume 2 µL; flow rate 0.313 mL/min; and column temperature 28 °C. The mobile phases were (i) H_2_O with 1% MeCN and 1 ppm guanidine hydrochloride (Pismennõi et al. 2021) and (ii) MeCN with 1% H_2_O and 1 ppm guanidine hydrochloride. The gradient program for LC separation was 0 to 10 min linear gradient 10% to 25%A, 10 to 12 min hold at 25%A, 12 to 14 min linear gradient 25% to 35%A, 14 to 14.5 min hold at 35%A. The MS parameters were set as negative ionization; source temperature 120 °C; drying gas flow 8 L/min; nebulizer pressure 35 psi; sheath gas temperature 120 °C; sheath gas flow 11 L/min; capillary voltage 2300 V; nozzle voltage 1000 V; fragmentor 175 V; and MS range 100 to 1100 *m*/*z*.

Single ion chromatograms were extracted for [M + Cl]^−^ adducts at *m*/*z* 215.0328 (glucose and fructose), 217.0484 (mannitol), 221.0529 (glucose ^13^C_6_), 229.0484 (pinitol), 377.0856 (saccharose and trehalose), and 539.1384 (raffinose). To convert the area under the peak into concentration, we constructed a calibration curve for each standard sugar using Agilent MassHunter Quantitative Analysis for Q-TOF from a standard solution containing each of the sugars and the internal standard. The concentration of sugars in each sample was expressed as mass of sugar (mg) per gram dry weight (mg·g dw^−1^).

Starch digestion and analysis were conducted using a previously published protocol (Bellasio et al. 2014). Briefly, the digestion was carried out in 2 sequential steps; the first included incubating the plant pellet (previously freed of soluble sugars) with α-amylase from *Bacillus licheniformis* (Megazyme—3,000 U/mL) at 100 °C, followed by incubation with amyloglucosidase from *Aspergillus niger* (Megazyme—3,260 U/mL) at 50 °C. The resulting glucose subunits from starch digestion were then measured by incubating the final diluted solution with a peroxidase–glucose oxidase preparation (Sigma #P7119–1 capsule/100 mL of water) and an o-dianisidine solution (Sigma #D3252—2.5 mg/mL) for 45 min in the dark, followed by a H_2_SO_4_ treatment, and finally an absorbance reading at 530 nm on a UV–VIS spectrophotometer. The obtained starch concentrations were expressed as milligrams per gram dry weight (mg·g dw^−1^).

### Statistical analysis of phenology

Ordered probit regressions were used to predict the probability of observing a phenological phase of apical and lateral buds (i.e. Phases 1 to 6), considered as a qualitative ordinal variable (Deslauriers et al. 2019):


$$P(E_{ij},t)=\;\frac{1}{1+\exp(t\cdot b_{DOY}+b_{\;phase})},$$

where *P* represents the probability of observing a given phenological phase (*E_i_*, from 1 to 6), for a given *j* tree at a given time *t*, expressed in DOY, and *b* represents the values of the model parameters. In this ordinal regression model, all *E_i_* phases share a similar slope. The relationship between the probabilities of the response variable [P(*E_ij_,t*)] and the linear predictors (time, expressed in DOY) was determined using the *polr* function in the R [version 4.3.2 (R Core Team 2023)] package MASS (Venables and Ripley 2002). We then used ordered probit regression to determine the DOY at which there was 50% probability of transition for each *E_i_* phase for all *j* trees. Phases 1 and 6 were considered as the beginning and end of phenology, respectively.

The *polr* function of the MASS package (Venables and Ripley 2002) was also used to test the effect of species (*Sp*), warming (*W*), and defoliation (*D*) on the phenological phase (E*_i_*) of a *j* tree according to


$$P(E_{ij},t)=\;\frac{1}{1+\exp\;(t\cdot b_{DOY}+b_{Ei}+b_{Sp}+b_{W}+b_{D}+b_{Sp\;\times W\;}+b_{Sp\;\times \;D}+b_{W\;\times \;D}+b_{Sp\;\times \;W\;\times \;D})},$$

where $P(E_{ij},\;t)$ represents the probability of observing a particular phenological stage *E_i_* at a given time *t* (in DOY). Several arguments of the *polr* function were tested (i.e. logistic, probit, and loglog) to compare the phenology of apical and lateral buds among linear predictors. The relationship between the probabilities of the response variable (number of monitored buds at a certain stage at a certain time) and the linear predictors (DOY, species, warming, and defoliation) was determined using the option “logistic.” To provide more robust estimates and reduce variability, we applied a bootstrap resampling technique using the *modelr* package (version 0.1.11) in R (Wickham 2023). Differences between species, warming, and defoliation were found using the LSMEANS option using a Bonferroni multiple test comparison (Deslauriers et al. 2019).

### Statistical analysis of allometry

We ran Pearson correlation to assess the relationship between the allometric traits and the beginning (Stage 1), end (Stage 6), and duration of the phenological stages. The effect of species, warming, and defoliation (i.e. the fixed factors) was tested on the branches' allometric traits through multivariate analysis of variance (Wilds' λ, MANOVA, run in PROC GLM, SAS 9.4, SAS Institute, Inc., Cary, NC). The allometric traits were used as multiple dependent variables (*y*), with the residual as the error term. A canonical discriminant analysis (run in PROC CANDISC, SAS 9.4) provided a canonical correlation between the quantitative variables and a fixed factor as a classification dummy variable. Only significant fixed factors and their interactions given by MANOVA were used. Then, Pearson correlation between the canonical variables (Can*_i_*) and the quantitative variables served to determine their importance in defining Can*_i_*. Linear regressions of Can*_i_* and phenology allowed for the inferring of the influence of allometry on phenology.

### Statistical analysis of sugars

Given that the progression of phenology was specific to each plant, species, and warming and defoliation treatment, the concentration of sugars and starch was inferred for each phenological phase (*Ei*, from 1 to 6). Thus, for each tree and analyzed organ (needles, twigs, and developing buds), the quantity of sugar at each phenological phase (*Ei*, from 1 to 6) was inferred using local polynomial regression fitting. This analysis was performed with the *loess* function from the stats package in R. The *loess* function was specified with a span parameter of 0.75 and a degree parameter of 2. This configuration implies a quadratic fit where 75% of the data points in the surrounding area are used for each local fit, aiming for producing a smooth overall fit.

Mixed models were used to determine the effect of organs, species, warming, and defoliation on sugar and starch concentrations across the 6 phenological phases (*Ei*, from 1 to 6) using both PROC GLIMMIX and PROC MIXED (SAS). A top-down strategy was used by first adding all fixed effects and their interactions and testing a set of random effects (West et al. 2022). The need to include random effects and a heterogeneous variance term was tested using the COVTEST option (GLIMMIX, SAS 9.4). A random intercept with *tree nested in plot* as subject was retained as a random effect.

## Supplementary Material

kiaf435_Supplementary_Data

## Data Availability

Sugar, allometry and phenological datasets are available at: https://doi.org/10.5683/SP3/RU54ZJ.

## Supplementary data

The following materials are available in the online version of this article.

**Supplementary Figure S1.** Allometric traits with new (2019) and older (2017 to 2018) internodes including number of needles (*N*), SLA (m^2^·kg^−1^), and wood volume (cm^3^).

**Supplementary Figure S2.** Mean sugar and starch concentrations (mg·g dw^−1^) from mid-April to mid-June in balsam fir and black spruce.

**Supplementary Figure S3.** NSC concentrations ± Sd (mg·g dw^−1^) during bud-opening phases in needles, buds, and shoots.

**Supplementary Figure S4.** Transport illustration of sucrose and D-pinitol from older foliage to new shoot growth during bud opening with growing cell illustration forming a carbon sink or metabolites pool in either cytosol or vacuole

**Supplementary Figure S5.** T-FACE plot disposition installed at the Valcartier Forest Research Station and illustration of the analyzed allometric traits.
