# Supplementary material for: The effects of metabolic and functional traits on bud opening: Comparing warming and defoliation in conifers
Source: Plant Physiol. 2025 Sep 26;199(2):kiaf435. doi: 10.1093/plphys/kiaf435 (PMC12526955; doi:10.1093/plphys/kiaf435)
Supplement: kiaf435_Supplementary_Data [file kiaf435_supplementary_data.pdf]

Supplementary Material

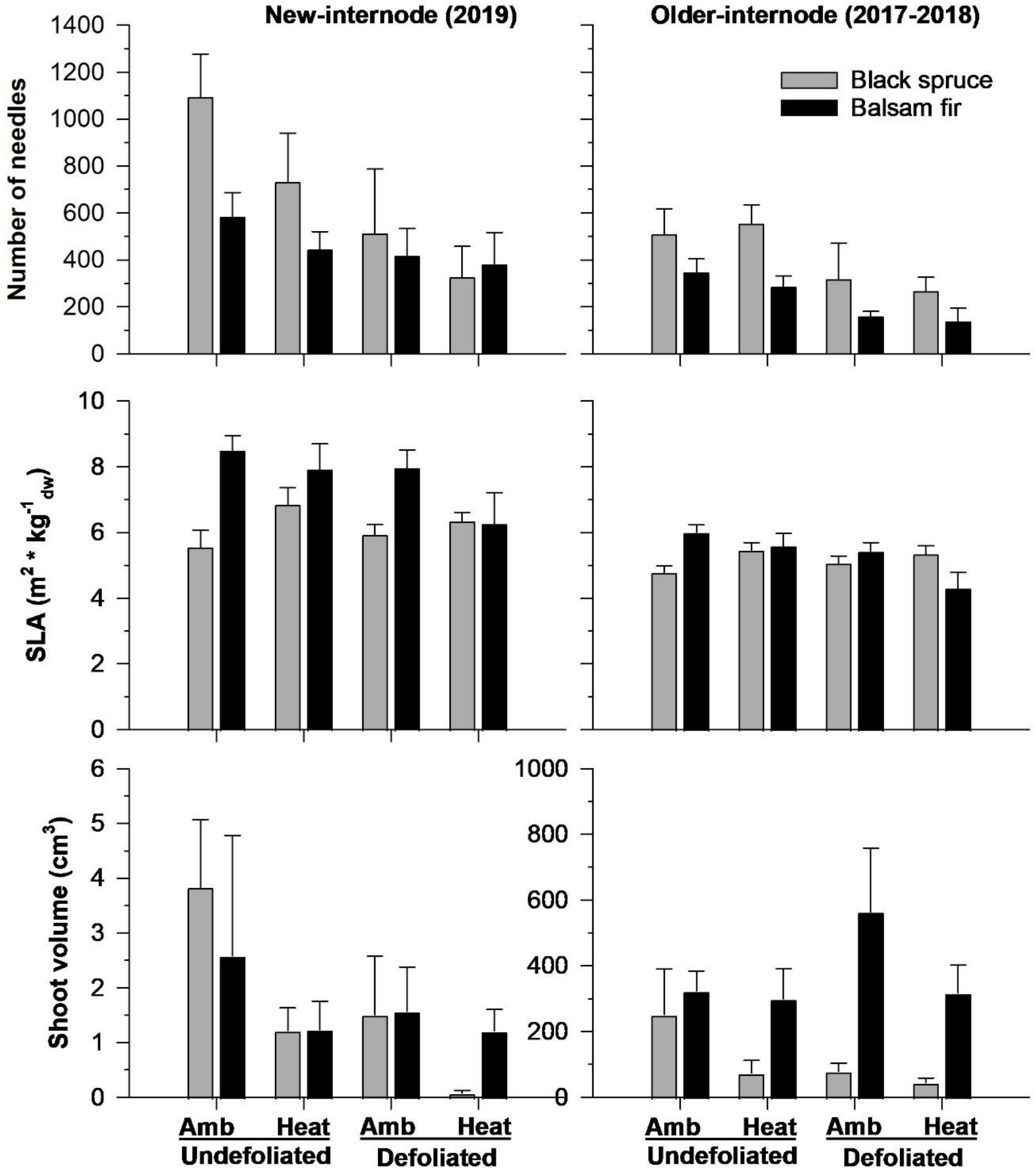

Supplementary Figure S1. Allometric traits including new (2019, left column) and older (2017–2018, right column) internodes including number of needles (*N*), surface leaf area (SLA, $m^2{\cdot}kg^{-1}$), and wood volume (cm^3). Vertical bars indicate standard error of the mean for each species (black spruce and balsam fir), warming (ambient or heating), and defoliation (undefoliated or defoliated) trees.

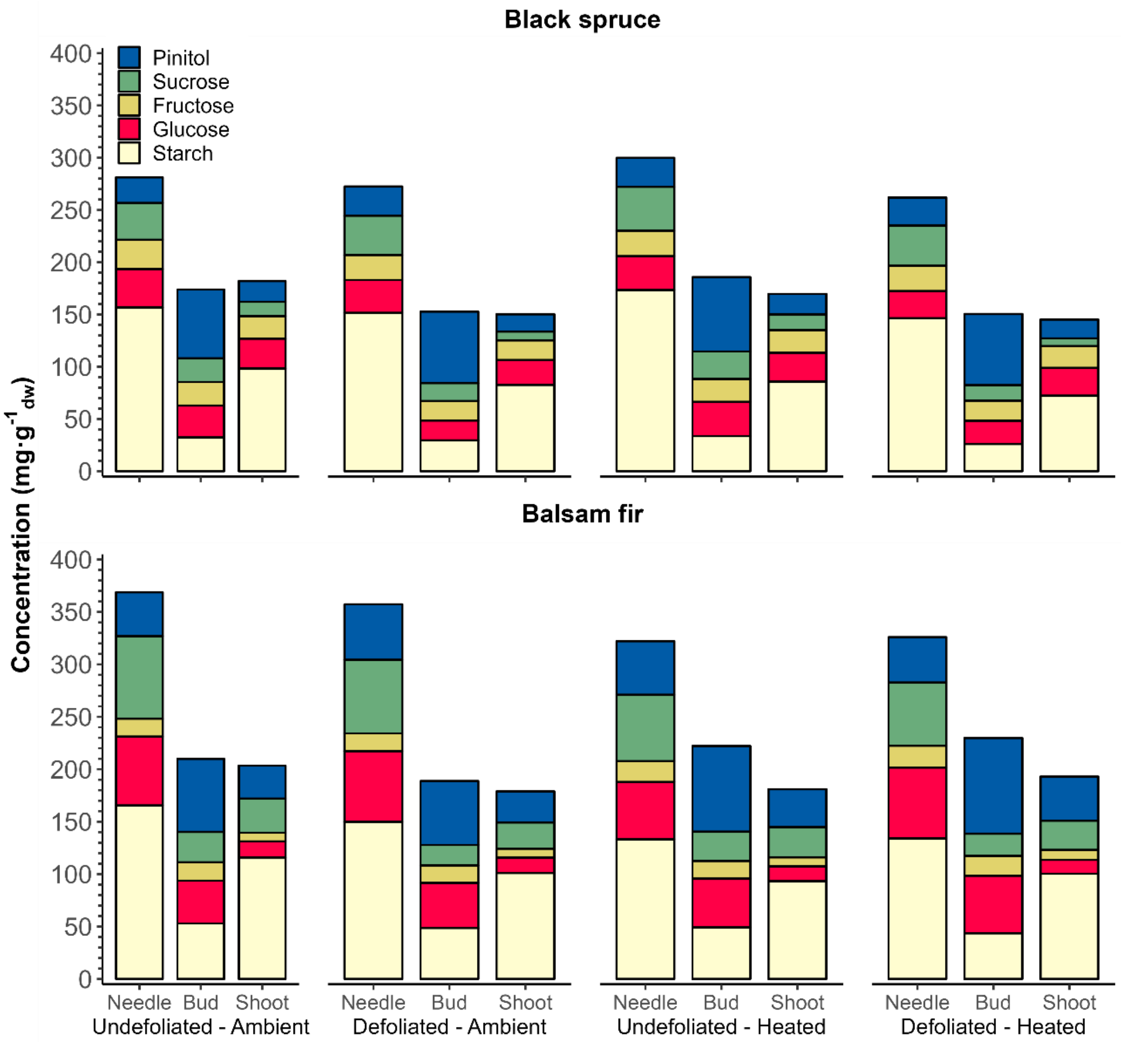

Supplementary Figure S2. Mean sugar and starch concentrations (mg·g dw^{-1}) from mid-April to mid-June in balsam fir (*upper row*) and black spruce (*lower row*). Measurements were performed on needles, buds, and shoots, for all experimental (defoliated/heated) and control (undefoliated/ambient) trees. The sugars correspond to pinitol (blue), sucrose (green), fructose (orange), glucose (red) and starch (off-white).

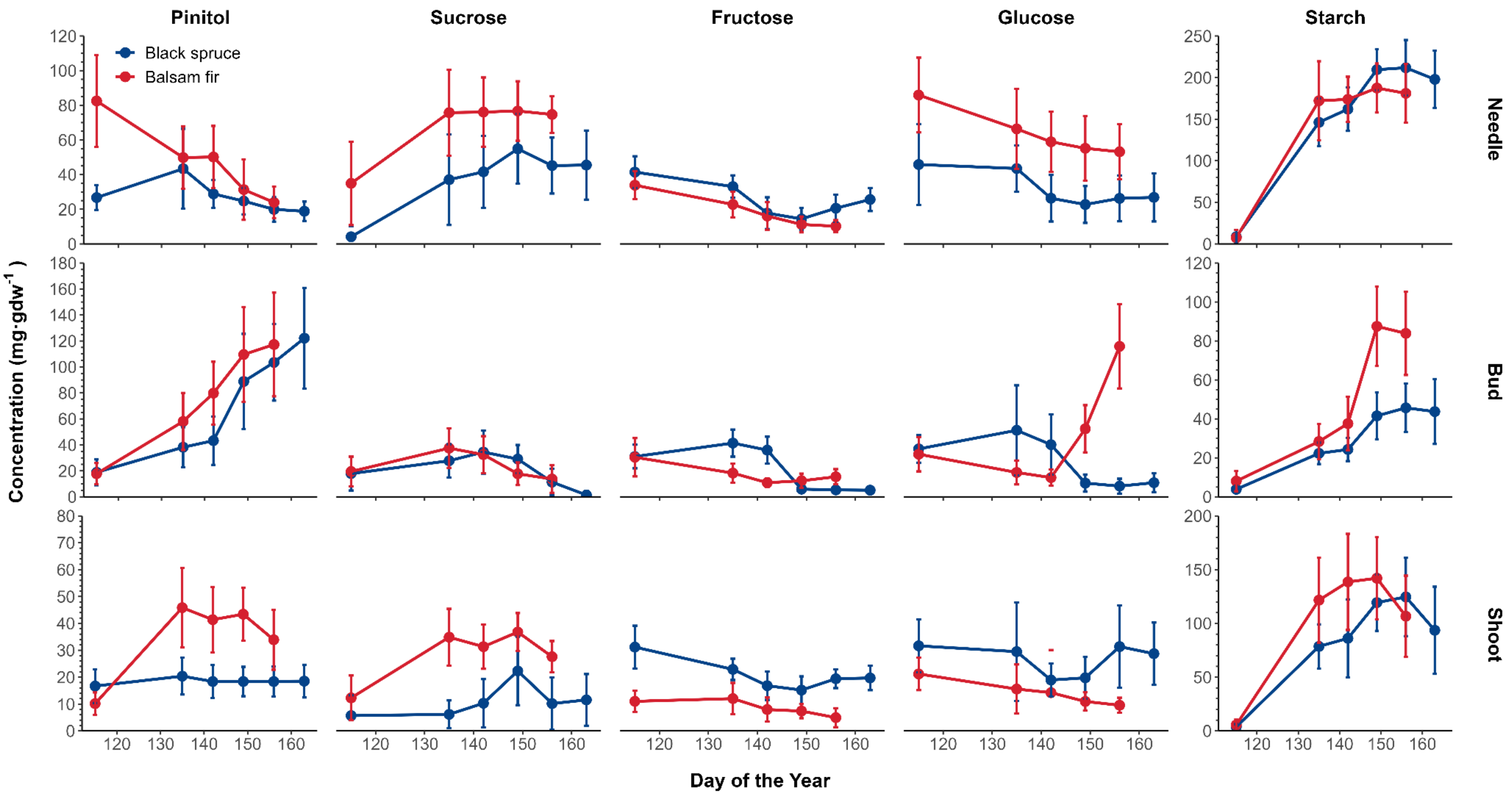

Supplementary Figure S3. Non-structural carbohydrate (NSC) concentrations ± standard deviation (mg·g dw^{-1}) during bud-opening phases in needles, buds, and shoots. The NSC includes glucose, fructose, pinitol, sucrose, and starch for black spruce (in blue) and balsam fir (in red).

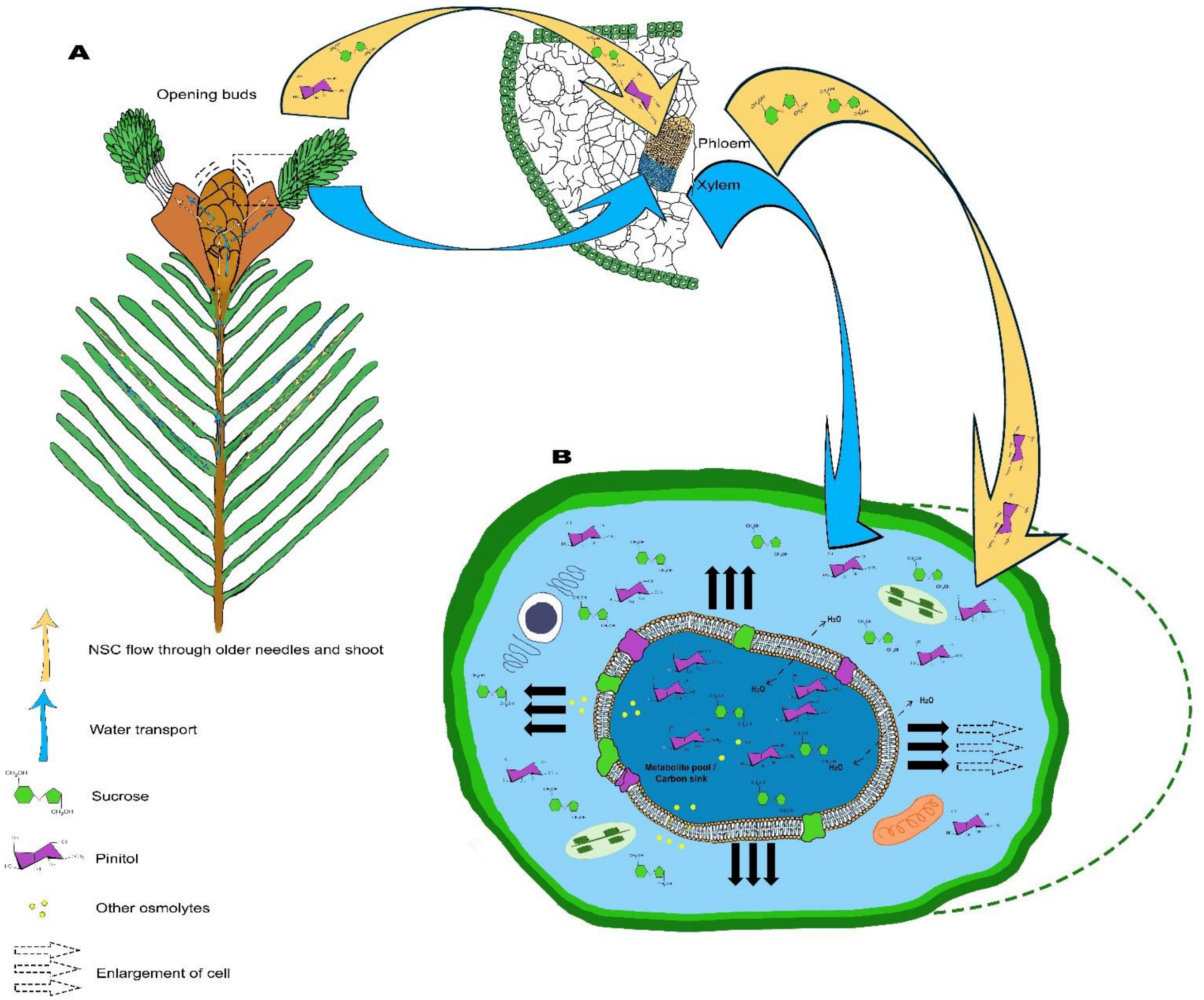

Supplementary Figure S4. A- Transport illustration of sucrose and D-pinitol from older foliage to new shoot growth during bud opening. **B-** Growing cells illustration with osmolytes, such as pinitol, forming a carbon sink or metabolites pool in either cytosol or vacuole. This pool generates and/or maintains water influx for bud swelling and shoot growth.

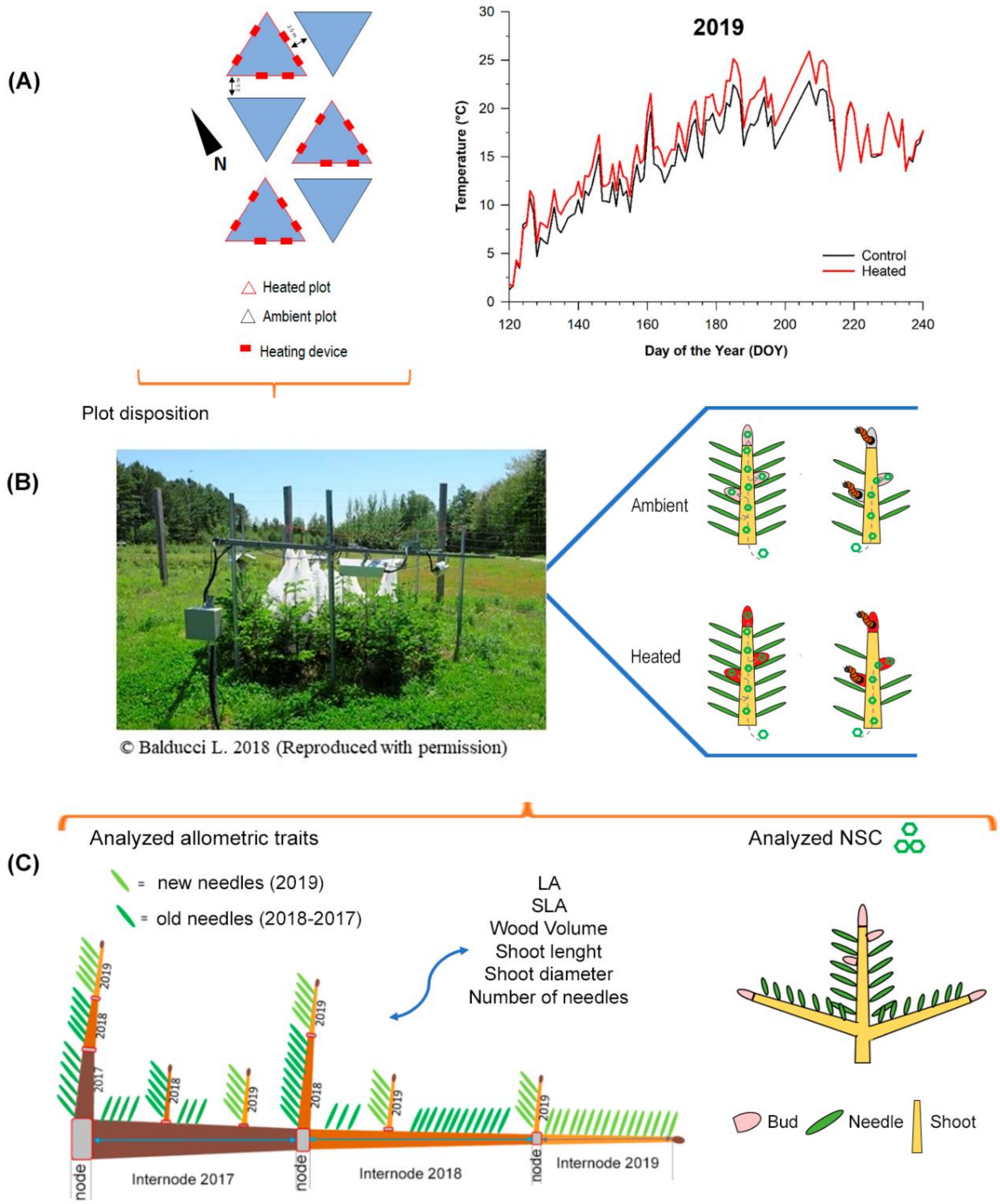

Supplementary Figure S5. A- Plot distribution, red borders represent heated plots, blue triangles represent ambient plots. Mean daily temperature variation from May to August (expressed in DOY) in ambient and heated treatments in 2019. **B**- T-FACE plot disposition installed at the Valcartier Forest Research Station (Natural Resources Canada). The white textile net covered each tree to protect the surrounding environment from spruce budworm (SBW) escape, each plot had defoliated and non defoliated individuals. **C**- Analyzed allometric traits, SLA, LA, wood volume, shoot length, shoot diameter and number of needles were studied from new needles (2019) and old needles (2017-2018) along with internodes (2017-2019). NSCs where obtained from apical and lateral buds, needles and shoots of 2019 new- internodes and 2017-2018 older-internodes.
